# Crosstalk between circadian clocks and pathogen niche

**DOI:** 10.1371/journal.ppat.1012157

**Published:** 2024-05-09

**Authors:** Helene Borrmann, Filipa Rijo-Ferreira

**Affiliations:** 1 Berkeley Public Health, Molecular and Cell Biology Department, University of California Berkeley, Berkeley, California, United States of America; 2 Chan Zuckerberg Biohub–San Francisco, San Francisco, California, United States of America; Joan and Sanford I Weill Medical College of Cornell University, UNITED STATES

## Abstract

Circadian rhythms are intrinsic 24-hour oscillations found in nearly all life forms. They orchestrate key physiological and behavioral processes, allowing anticipation and response to daily environmental changes. These rhythms manifest across entire organisms, in various organs, and through intricate molecular feedback loops that govern cellular oscillations. Recent studies describe circadian regulation of pathogens, including parasites, bacteria, viruses, and fungi, some of which have their own circadian rhythms while others are influenced by the rhythmic environment of hosts. Pathogens target specific tissues and organs within the host to optimize their replication. Diverse cellular compositions and the interplay among various cell types create unique microenvironments in different tissues, and distinctive organs have unique circadian biology. Hence, residing pathogens are exposed to cyclic conditions, which can profoundly impact host–pathogen interactions. This review explores the influence of circadian rhythms and mammalian tissue-specific interactions on the dynamics of pathogen–host relationships. Overall, this demonstrates the intricate interplay between the body’s internal timekeeping system and its susceptibility to pathogens, which has implications for the future of infectious disease research and treatment.

## 1. Background on circadian rhythms in mammals

**Fig 1:**
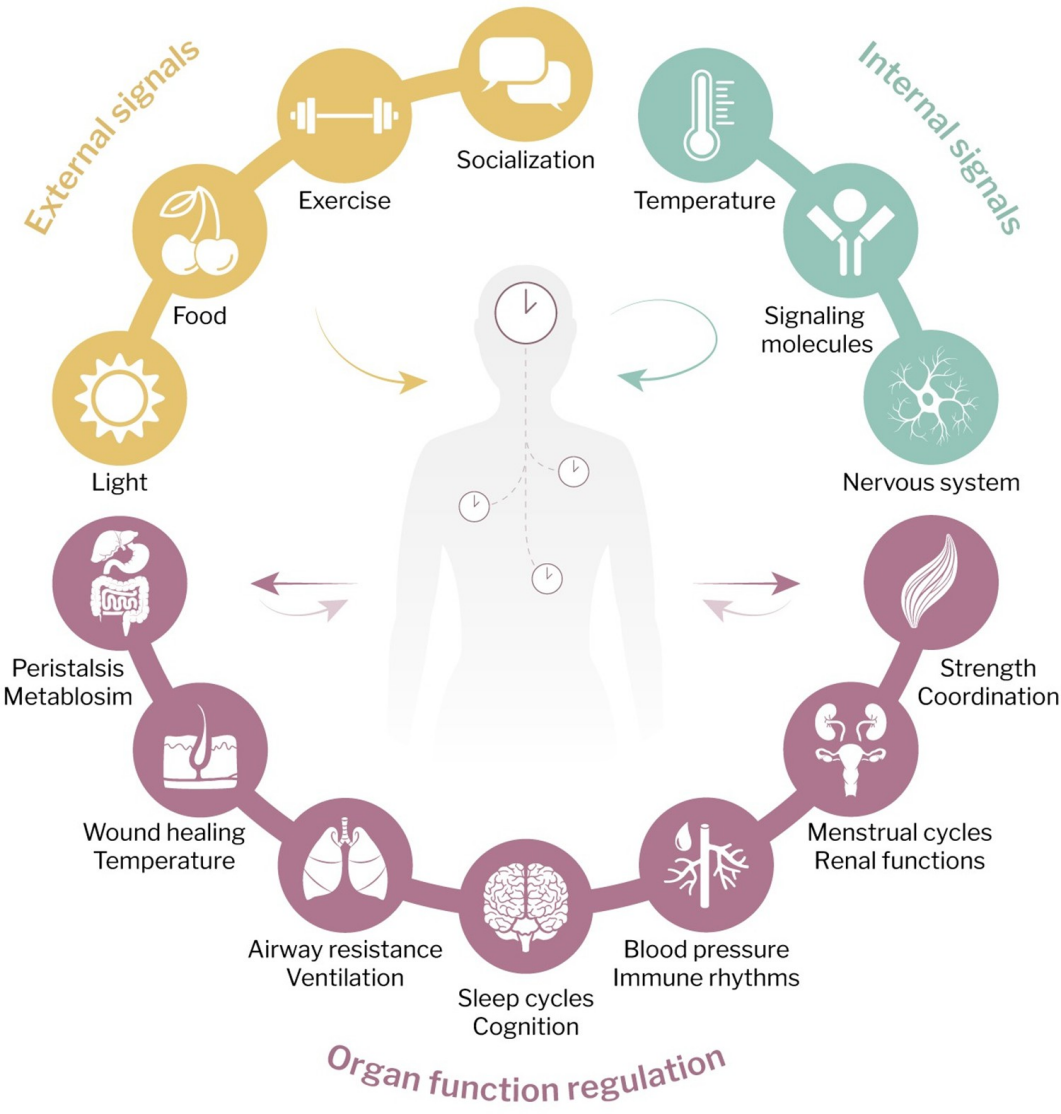
Circadian regulation of the body. Circadian rhythms impact diverse physiological processes across the body. The central clock in the brain integrates external and internal signals and communicates with tissue clocks to regulate organ functions. These include the nervous and endocrine system [4], cardiovascular system [1], respiratory tract [95], digestive system [61], reproductive [119] and urinary system [128], muscle skeletal system [2], and skin [111].

The Earth’s rotation governs day and night cycles, which shapes the evolutionary adaptation of all organisms on our planet to these rhythmic environmental variations. Circadian rhythms are 24-hour cycles that are widely observed across all domains of life. In mammals, the circadian system operates in a hierarchical framework, including central, peripheral, and molecular clocks. The suprachiasmatic nucleus (SCN) in the brain is the central pacemaker that integrates systemic cues, for instance, from light through the retinohypothalamic tract. It processes internal signals and synchronizes peripheral clocks in different tissues, causing daily changes in physiology and organ functions [1–4] (**Fig 1**).

**Fig 2:**
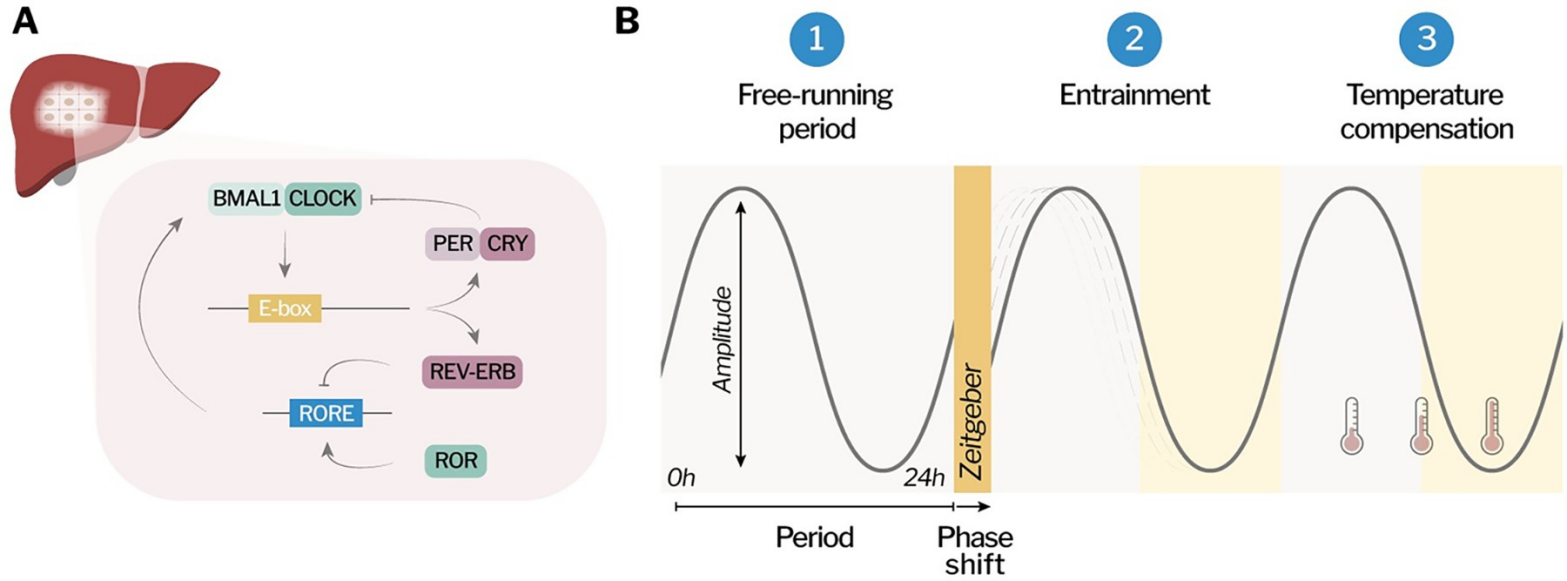
Molecular clock and circadian criteria. **(A)** Every cell in the body has an intrinsic circadian clock, in which transcriptional/translational feedback loops coordinate rhythmic gene expression. The activators BMAL1:CLOCK bind to E-boxes in promoter regions of genes and activate the expression of REV-ERB, ROR, PER, and CRY. PER:CRY inhibit BMAL1 activity, and ROR and REV-ERB compete for binding to RORE’s, including in the *Bmal1* promoter. The figure represents 2 out of the 3 interconnected loops that regulate the clock, focusing on those that have been connected to host–pathogens interactions. **(B)** Criteria for biological rhythms to classify as circadian rhythms: (1) endogenous free-running period of approximately 24 hours; (2) entrainment to cues called Zeitgebers, for instance, light pulse causing phase shift; and (3) temperature compensation.

At the molecular level, each cell has a clock [5] that is governed by self-sustained transcriptional translational feedback loops that generate oscillations in gene expression (**Fig 2A**) [6]. The 2 main activators BMAL1 (brain and muscle ARNT-like 1) and CLOCK (circadian locomotor output cycles kaput) heterodimerize and bind to genomic elements called E-boxes, thereby activating the expression of circadian regulated genes, including their own repressors. Period (PER1/2/3) and Cryptochrome (CYR1/2) form a repressive complex that inhibits the activity of BMAL1 and CLOCK, creating a negative feedback loop [7]. A second interlocked feedback loop comprises the repressors REV-ERBα/β and activators RORα/β/γ (RAR-related orphan receptors), which themselves contain E-boxes in their promoters and are regulated by BMAL1 and CLOCK. REV-ERBs and RORs compete for binding to ROR response elements (RORE) in regulatory regions of genes, including the *Bmal1* promoter [6,8]. BMAL1/CLOCK further regulate the expression of PAR-bZip transcription factors (proline and acidic amino acid-rich basic leucine zipper), which bind to D-box elements and form a third arm of the cellular clock. These factors include DBP (D-box binding protein), TEF (thyrotroph embryonic factor), and HLF (hepatic leukemia factor) that interact with the REV-ERB/ROR-driven repressor NFIL3 (nuclear factor interleukin-3 regulated). In addition to transcriptional regulation, posttranscriptional and posttranslational cellular processes contribute to circadian oscillations [9,10]. Cellular rhythms can also persist without transcription, such as the oxidation–reduction cycles of peroxiredoxin proteins [11,12].

The term “circadian” derives from “circa” and “diem,” Latin for “about” and “a day,” but it’s important to note that not all daily rhythms are considered circadian. To qualify as a circadian rhythm, a biological pattern must meet the following 3 criteria (**Fig 2B**): (1) It must have an endogenous free-running period of approximately 24 hours, which is maintained in the absence of environmental cues. (2) Circadian oscillations can be entrained by exposure to external stimuli, called Zeitgebers, that cause readjustment of the timing (phase and period) of the oscillations. (3) Circadian periodicity is temperature compensated, hence maintained over a range of physiological temperatures [13]. Circadian rhythms provide organisms with the ability to predict and adjust to recurrent environmental variations. This enables optimized resource utilization, including light and food, thereby enhancing their evolutionary fitness [14–17]. There is increasing evidence that pathogens that infect mammals are regulated through circadian mechanisms of their hosts or have intrinsic clocks themselves [18–20].

## 2. The importance of host environments for infections

**Fig 3:**
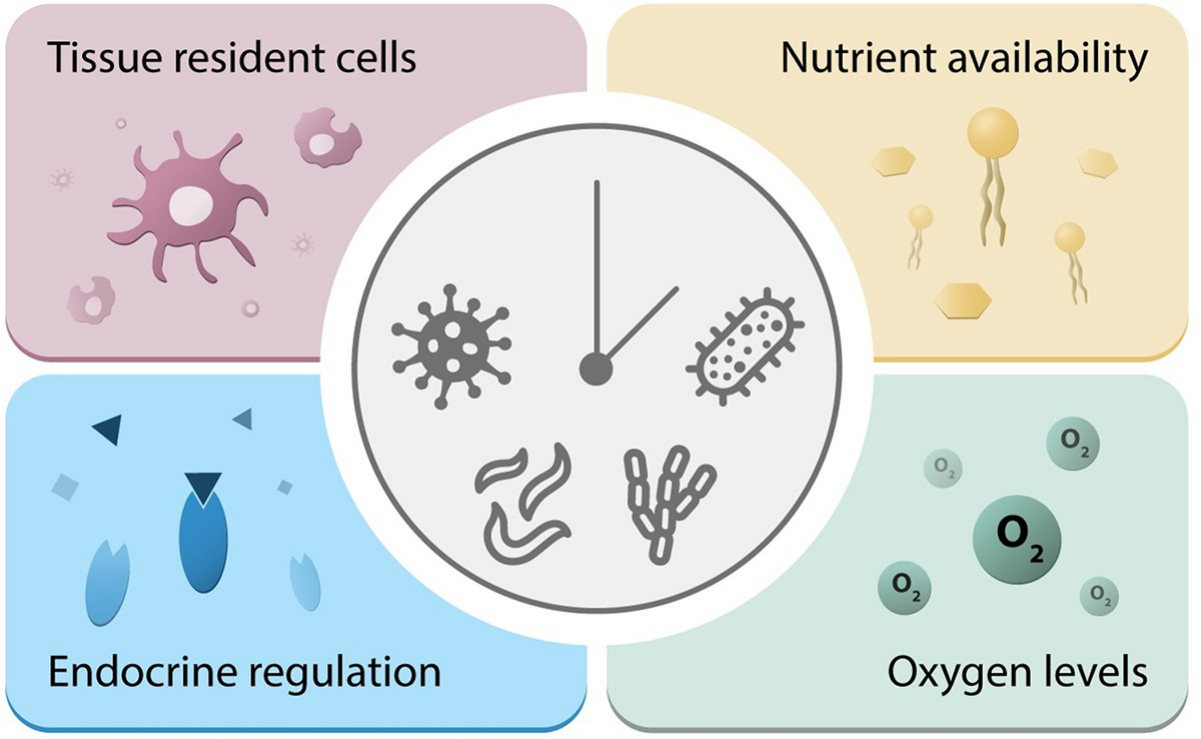
Pathogens are influenced by the host’s microenvironments. Bacteria, parasites, viruses, and fungi have evolved to maximize their colonization, replication, and transmission in specific niches within hosts. Multiple factors can vary depending on the infected organ. These include nutrient availability, variations in oxygen levels, endocrine regulation, and the presence of tissue-resident cells. The circadian clock can influence all these elements.

Many pathogens, including bacteria, viruses, parasites, and fungi, exhibit dependency on hosts for survival. Through evolutionary adaptations, they have fine-tuned their replication strategies to maximize efficiency within specific niches in hosts. Variations in host microenvironments can either facilitate or hinder pathogen colonization, replication, and transmission, thus critically impacting disease outcomes [21,22]. These environments encompass a diverse interplay of physiological, immunological, and microbiological factors (**Fig 3**).

Circadian rhythms are important in shaping organ-specific niches, for instance, by regulating differences in nutrient availability depending on the time of the day [23,24]. While this is primarily a mechanism to ensure optimal organ function, pathogens that infect the tissue may have an advantage from adopting and utilizing this cyclic nutrient availability. Specialized immune cells, such as tissue-resident macrophages and dendritic cells, serve as a first line of defense against pathogens entering specific tissues [25]. For instance, alveolar macrophages in the lung act against inhaled pathogens [26] and microglia in the brain play a crucial role in protecting the central nervous system from infections [27]. It has been shown that multiple arms of the immune system are under circadian regulation and that immune cell intrinsic clocks are important for an effective response to pathogens [28,29]. Around 8% of the transcriptome from murine peritoneal macrophages is under circadian regulation [30], and deletion of *Bmal1* in T cells reduced rhythmic cell-intrinsic receptor expression in mice [31]. Moreover, the rhythmic recruitment of immune cells to tissues [32,33] can influence temporal regulation of the protection against pathogens in organs. Loss of *Bmal1* reduced rhythmic recruitment of myeloid cells to arteries and veins [34]. Additionally, *Bmal1* knockout (KO) increased dendritic cell migration into skin lymphatic vessels during the morning in mice [35]. Thus, circadian fluctuations in immune responses may differ among distinct organs, emphasizing the role of circadian regulation in host environments as an important factor in the context of infections.

Selective pressures often drive the emergence of new pathogen variants and strains with distinct virulence profiles and host specificities. Therefore, a comprehensive understanding of the intricate relationships within host environments, including circadian rhythms, is indispensable for advancing our strategies for infectious disease prevention and treatment.

## 3. Interplay between clocks and pathogens in different host niches

**Fig 4:**
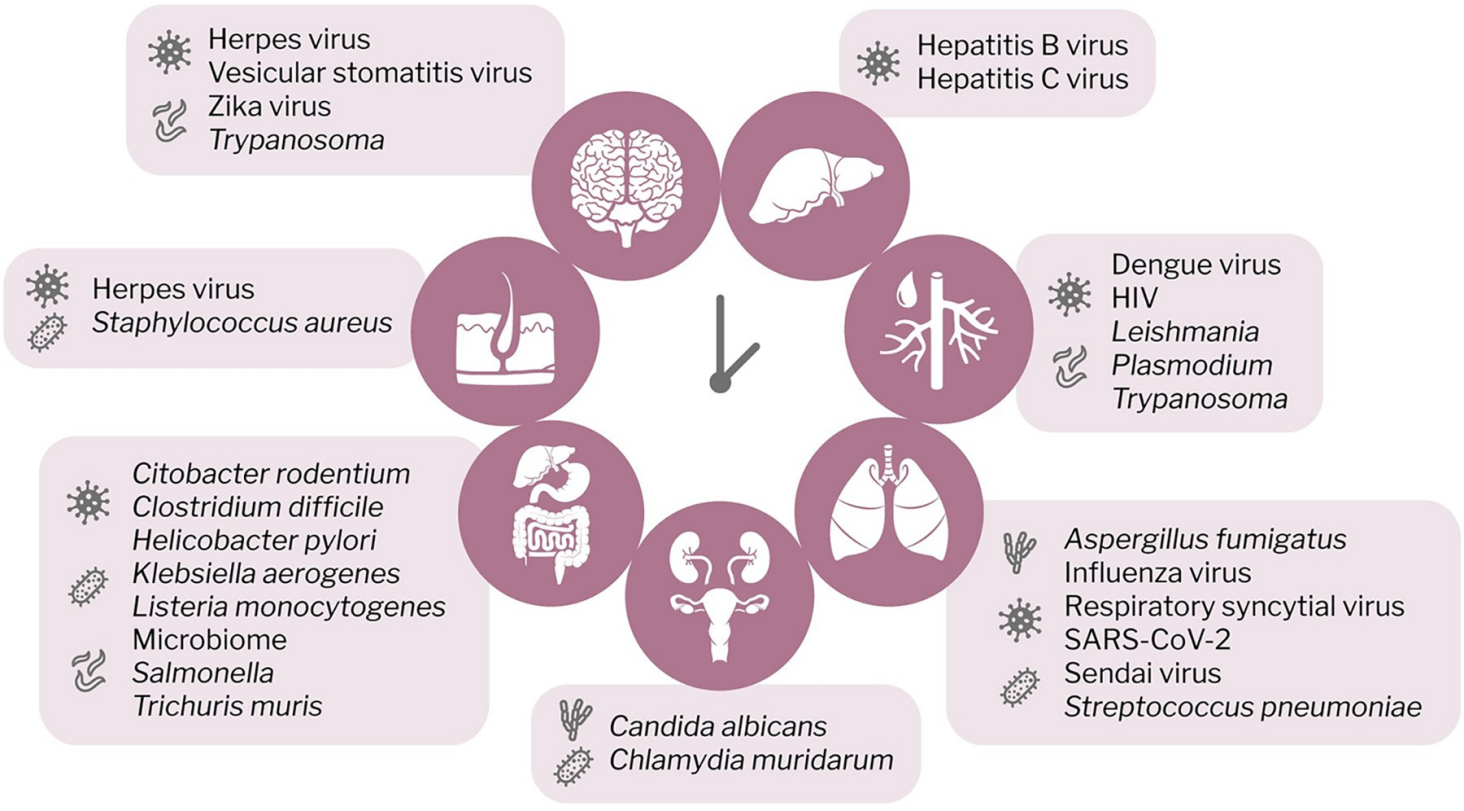
Interplay between body clocks and pathogens. Different pathogens target specific organs for replication, and some are capable of infecting multiple organs at various life stages. An interplay with circadian rhythms has been demonstrated for the pathogens shown. Liver: hepatitis B virus [44,45,51], hepatitis C virus [46,48, 50]. Blood: dengue virus [46], HIV [74–77], *Leishmania* [78], *Plasmodium* [86–90], *Trypanosoma* [91,92]. Respiratory tract: *Aspergillus fumigatus* [109,110], influenza virus [31,97–100], respiratory syncytial virus [104], SARS-CoV-2 [101–103], Sendai virus [98], *Streptococcus pneumoniae* [107,108]. Reproductive and urinary system: *Candida albicans* [129], *Chlamydia muridarum* [124–126]. Gastrointestinal tract: *Citobacter rodentium* [71], *Clostridium difficile* [70], *Helicobacter pylori* [31], *Klebsiella aerogenes* [65], *Listeria monocytogenes* [72], microbiome [53,62,67], *Salmonella* [68,69], *Trichuris muris* [73]. Skin: herpes virus [97,115–117], *Staphylococcus aureus* [114]. Brain: herpes virus [131], vesicular stomatitis virus [135], Zika virus [46], *Trypanosoma* [133].

Circadian rhythms manifest within host tissue environments, and numerous pathogens target comparable host niches, thereby experiencing similar circadian environments. Here, we will summarize the literature advances in the field over the past years, mostly focusing on each pathogenic infection when encountering the tissue (**Fig 4**). However, it is likely that infections also change the circadian regulation of the tissue and thus would be interesting to study it in the context of coinfections in the future.

### The liver

The liver is the body’s largest solid internal organ and plays a central role in metabolism, detoxification, and the synthesis of essential proteins. Various cell types in the liver, including hepatocytes, sinusoidal endothelial cells, and Kupffer cells, cooperate spatiotemporally to shape and maintain liver functions [36]. A highly organized architectural pattern and vascularization receiving blood from both the hepatic artery and portal vein results in liver zonation. It causes specific microenvironments with varying oxygen tensions, nutrients, and toxins [37,38], which can influence colonization by pathogens.

The liver is one of the most circadian regulated organs with 20% of the transcriptome showing rhythmic expression [39,40], which impacts various of its functions [41,42]. Circadian disruption results in dysregulation of bile acid homeostasis in the liver [43], and transcription of the transmembrane mediator of bile acid transport (sodium taurocholate cotransporting polypeptide, NTCP) is regulated by the clock transcription factors REV-ERBα and β [44]. Interestingly, NTCP is required for entry of hepatitis B virus (HBV) into cells, and HBV is further regulated by the circadian machinery through BMAL1 binding to the viral promoter [44]. Similarly, entry of hepatitis C virus (HCV) into hepatocytes is regulated by BMAL1, and *Bmal1* silencing or activation of REV-ERBα/β with synthetic agonists inhibits the replication of HCV in vitro. Lipids are essential for generation of new viral particles, and REV-ERBα was shown to regulate lipid and cholesterol metabolism [45]. Consistently, activation of REV-ERBα reduced expression of stearoyl-CoA-desaturase, a rate limiting enzyme for HCV replication [46]. Circadian rhythms and liver metabolism are connected through peroxisome proliferator-activated receptors (PPAR), which are rhythmically expressed, and control glucose and lipid metabolism [47]. Liver metabolism was shown to be disturbed in HCV-related cirrhosis through deregulation of BMAL1 [48]. Hepatic viruses can promote the formation of hepatocellular carcinoma, and the expression of *Per1-3* and *Cry2* was reduced in this tissue, which indicates that the crosstalk is bidirectional [49–51]. Interestingly, the initiation and progression of virus-related liver cirrhosis was partially regulated by the gut microbiota through the gut–liver axis [52], and microbiota regulated the circadian liver transcriptome and detoxification pattern [53].

Malaria infection of the host is initiated in the liver where *Plasmodium* parasites replicate during various days, but there is limited knowledge about the circadian regulation of this phase in the parasite life cycle. Hypoxia promotes liver-stage malaria infection [54], and an interplay of hypoxic and circadian pathways has been described [55,56], raising the question if this could influence hepatic *Plasmodium* replication. Neutrophils were shown to infiltrate the liver in a time-of-day–dependent manner in uninfected mice, where neutrophil numbers peaked at the onset of the rest phase and were lowest at the beginning of the night [57]. Neutrophils are known to be important for the response to malaria [58], and while rhythmicity in neutrophil infiltration could result in daily oscillations of parasites in the liver, further studies are required to validate these hypotheses.

### The gastrointestinal tract

The gastrointestinal tract is formed by various segments including the stomach, small intestine, and large intestine and functions as an interface connecting the external environment with the body. Its multifaceted role, encompassing digestion and protection, is evident in its distinctive microenvironments. These include high acidity, which aids in the digestive process, the presence of microvilli to enhance nutrient absorption, and the formation of gut-associated lymphoid tissue through tissue-resident immune cells. Additionally, the commensal gut microbiome, a complex community of microorganisms, contributes significantly to metabolism and highlights the importance of microbes for overall gut health [59,60]. Multiple metabolic processes are regulated by circadian rhythms, and metabolic signals provide feedback to the circadian timing system [24,61].

The intestinal microbiome exhibits daily oscillations [62] and levels of bacteria colonizing the epithelial layer were up to 10-fold higher during the active phase compared to the rest phase in mice [53]. Because these microbiome rhythms are almost absent in circadian clock mutant animals, there has not been a lot of research on the rhythms of the bacteria themselves. However, recent studies discovered that the commensal gut bacterium *Bacillus subtilis* has properties typically found in complex, multicellular circadian systems, including a free-running period, entrainment, and temperature compensation [63,64]. Similarly, the human gut bacterium *Klebsiella aerogenes* exhibited circadian rhythms in vitro, demonstrating entrainment to temperature cycles of different durations and amplitudes [65]. Rhythmic microbial and circadian cues are integrated by histone deacetylase 3, resulting in oscillations in histone acetylation, metabolic gene expression, and nutrient uptake [66]. Homeostasis in the intestinal epithelium is orchestrated by the circadian clock in conjunction with microbiota cues, which signal through rhythmic Toll-like receptor expression on intestinal epithelial cells [67]. Intestinal surface attachment of segmented filamentous bacteria in the mouse intestinal microbiota was shown to drive oscillations in innate immune response activation, resulting in periodic antimicrobial protein expression and rhythms in resistance to *Salmonella typhimurium* infection [68]. This is in line with findings of an increased proinflammatory response to *Salmonella enterica* and higher colonization levels when infection occurred during the rest period in mice [69].

The molecular clock of gut-resident immune cells, specifically type 3 innate lymphoid cells (ILC3s) was shown to influence circadian regulation of pathogens in the gastrointestinal tract. Mice with *Bmal1*-deficient ILC3s had more severe inflammation and higher bacterial burden of *Clostridium difficile* [70] and *Citrobacter rodentium* [71]. Moreover, clearance of *Listeria monocytogenes* was more effective when mice were infected intraperitoneally during the morning compared to the afternoon, which was regulated by diurnal oscillations of inflammatory monocytes [72]. The circadian immune system further contributed to the response against the rodent gut parasite *Trichuris muris*, with the circadian regulator BMAL1 governing the clock response in dendritic cells [73]. Mice were infected with *Helicobacter pylori* at 3 different times of the day and T cell counts in lymph nodes were quantified 3 weeks later. Cell numbers showed a circadian pattern with highest counts when infection occurred during the day, highlighting the role of circadian rhythms in modulating adaptive immune responses [31].

Generally, commensal and pathogenic gut bacteria are exposed to circadian rhythms in the gastrointestinal tract, and alterations in the gut microbiome can cause dysbiosis, which facilitates increased infections. This is likely to impact not only bacterial infections but also viruses, such as Norovirus, and parasitic infections like Giardiasis and Cryptosporidiosis in the gastrointestinal tract [19], which have not yet been explored within the circadian context.

### The blood

The importance of blood is obvious considering its components: red blood cells for gas exchange, white blood cells for immune defense, platelets for blood clotting and transport of nutrients, hormones, and waste products inside the plasma. As blood travels throughout the body, it encounters various organs and is exposed to different peripheral clocks. Additionally, blood is important for systemic circadian clock synchronization, as it transmits information through soluble messenger signals and daily variations in blood pressure and temperature. Just like all other cells in the body, blood cells have intrinsic circadian rhythms and can be infected by pathogens.

The main replication site of human immunodeficiency virus (HIV) are T cells, and it was demonstrated that clock transcription factors regulate viral gene expression [74]. This results in rhythmic viral replication in vitro [75] and in patients [76,77]. Macrophages and neutrophils can be infected by the intracellular parasite *Leishmania*. The susceptibility to *Leishmania* infection varied over 24 hours in mice, which was regulated by the circadian expression of chemokines and subsequent rhythmic infiltration of immune cells at the infection site. These rhythms of higher susceptibility to *Leishmania major* at night were abolished in mice with clock-deficient immune cells, indicating that immune cell clocks control the magnitude of *Leishmania* parasite infection [78]. Another pathogen infecting immune cells is dengue virus, and it has been shown that REV-ERBα/β agonists can reduce viral replication [46].

Red blood cells do not have nuclei, yet they possess intrinsic 24 hours rhythms through posttranslational modification of peroxiredoxins and redox changes in hemoglobin [79,80]. During their asexual stages, malaria parasites undergo replication within red blood cells, resulting in periodic rupturing of these cells. This process is responsible for the characteristic cyclic fevers that are a defining feature of the disease [81,82]. The temporal dynamics of malaria biology have been investigated by various studies [83–85], many of which found that the rhythmic behavior of *Plasmodium* is intricately governed by metabolic processes of the host [86–89]. Recent evidence shows that malaria parasites are not driven by the host’s circadian rhythms but rather possess their own intrinsic rhythms, which are synchronized to the host. Approximately 80% of parasite genes were expressed rhythmically with a 24-hour period, and oscillations were only reduced to approximately 60% of cycling genes in the absence of host rhythmic cues, demonstrating that rhythms in malaria parasites are, in fact, intrinsic [90]. However, the complex interplay and coordination of circadian rhythms between the parasite and host remain to be elucidated.

The causative agent of sleeping sickness, *Trypanosoma bruce*i, is another example of a blood-borne parasite under circadian regulation. In vitro culture revealed inherent circadian rhythms in *T*. *brucei* gene expression. Many of the parasite cycling genes were involved in metabolic pathways and parasite rhythms were not dependent on the cell cycle [91]. Interestingly, early studies found that *Trypanosoma* populations remained stable throughout the day in infected animals [92]. It is currently unknown whether there are circadian rhythms in infections of other blood-borne pathogens such as those that cause Chagas, Babesiosis, or Toxoplasmosis. Nonetheless, it is important to note that the timing of blood collection can impact the detection of immune markers and other pathogens [77], which should be considered in clinical settings.

### The respiratory tract

The respiratory tract encompasses complex structures extending from the nose down to delicate bronchioles in the lungs. It is lined with mucosal membranes and ciliated cells, which facilitate gas exchange between the body and the external environment. Early observations of daily fluctuations in lung function were made by ancient Greek physicians around 100 CE, who described that symptoms of asthma manifested at night [93]. These findings were confirmed, and later studies revealed that exacerbated asthma symptoms at night are regulated through the endogenous circadian system independent of sleep and other daily cycles [94]. Airways exhibit daily rhythms in caliber, being most constricted in the early morning (2 AM to 5 AM) when individuals with asthma are at a higher risk of experiencing respiratory symptoms. The heightened airway resistance coincides with an increased tolerance to CO_2_ buildup, likely reducing the effort required for breathing during sleep [95]. The respiratory tract is exposed to dynamic fluxes of microbial colonization and clearance, and upper and lower parts have a topographically distinct microbial composition [96].

Several pathogens infecting respiratory organs are regulated by circadian rhythms, one of the best studied of which is influenza virus. Replication of the flu virus was increased in arrhythmic *Bmal1* KO cells in vitro [97] and *Bmal1-*deficient mice in vivo [98]. Importantly, infection of mice at different time points did not result in changes in influenza viral load, and rhythms were conferred by rhythmicity in the immune response [31,99]. Infection at the start of the active phase promoted lung inflammation [99], further highlighting the importance of the tissue-specific environments on infection outcomes. Respiration largely defines the microenvironment in lungs, and, interestingly, exposure to neonatal excess oxygen abolished the clock-mediated time of day protection from influenza in mice. It was independent of viral burden, through host tolerance pathways, and loss of *Bmal1* in alveolar type 2 cells recapitulated increased mortality. This suggests that disruption is mediated by the lung’s internal clock, rather than central or immune systemic clocks [100].

Circadian disruption altered the lung transcriptome in mice, which predisposes animals to viral infection, including coronavirus SARS-CoV-2 [101]. SARS-CoV-2 enters cells through the angiotensin-converting enzyme type 2 receptor, which is expressed rhythmically and controlled by posttranscriptional circadian mechanisms. This causes variations in SARS-CoV-2 entry depending on the time of day. Additionally, the replication of various SARS-CoV-2 strains was reduced in *Bmal1* KO cells, which is likely regulated through an enhanced interferon-stimulated gene response [102,103]. The innate immune response in the lung further contributes to the circadian response to other viruses, for instance, pulmonary infection of *Bmal1* KO mice with respiratory syncytial virus [104] and Sendai virus [98] produced more severe disease outcomes than in wild-type (WT) animals.

Circadian regulation in the lung has been tested using lipopolysaccharides (LPS)-induced inflammation to mimic gram-negative bacteria infection. Interestingly, the circadian repressor REV-ERBα plays a central role in the regulation of pulmonary inflammation, involving both myeloid and bronchial epithelial cells [105]. In airway epithelial cells, the circadian variation in pulmonary inflammatory responses was shown to be independent of rhythmic glucocorticoid signaling [106]. Mice had higher antibacterial immunity when infected with *Streptococcus pneumoniae* during the active phase, and genetic ablation of *Bmal1* in bronchiolar cells disrupted rhythmic chemokine expression causing worse disease outcomes [107]. It was shown that BMAL1 inhibits macrophage motility and phagocytosis through regulation of a network of cell movement genes, thereby impairing the response against pneumonia in mice [108].

There is limited evidence regarding the circadian regulation of fungal infections in mammals; however, it has been studied in the context of the lung fungus *Aspergillus fumigatus*. In vitro, there was no variation in the phagocytic activity of macrophages over time, however, mice infected at night exhibited a 2-fold enhancement in lung clearance a few hours after the challenge compared with those inoculated in the morning [109]. Conversely, another study found that maximum colonization occurred when infection took place at the beginning of the active phase, when assessing fungal load at 1 or 3 days post-infection. This study further demonstrated that the circadian oscillation of tryptophan metabolism leads to diurnal changes in the immune response in the lung, regulating the outcome of *A*. *fumigatus* infection [110]. Variations in the timing of infection and organ collection between both studies underscore the importance of considering these timings in experimental designs.

### The skin

As the largest organ exposed to daily rhythms in sunlight and radiation, oscillations are observed in multiple cell types across all layers of the skin [111]. The skin is colonized with microbiota, which showed daily changes that correlated with human activity [112]. There are only a few studied examples of circadian regulated pathogens that infect the skin, including herpesvirus and *Staphylococcus aureus* [113]. Survival of *S*. *aureus* on mouse skin was reduced when infection occurred at the start of the active phase compared to the end [114]. Cutaneous herpes simplex virus 2 (HSV-2) infection was less severe when mice were infected during the rest phase compared to the active phase [115], and acute HSV-1 infection was enhanced in arrhythmic *Bmal1* KO mice [97]. On a molecular level, CLOCK interacts with viral proteins to remodel HSV chromatin [116] and was identified to be a component of the viral transcriptional complex [117].

Murine skin displays circadian-regulated expression of interferon-sensitive genes [118], and it is likely that this will affect the response to various other pathogens that infect the skin. Evidence is limited, but considering the circadian rhythms observed in the commensal microbiomes of other organs, it is plausible that a similar phenomenon holds true for the skin. Moreover, it is likely that circadian rhythms in the skin will not only affect direct pathogen infection but may also influence the invasion of pathogens transmitted by insect vectors.

### The reproductive and urinary system

The unique microenvironment in the reproductive tract undergoes distinct changes affecting factors such as pH, mucosal integrity, and hormone levels. These changes are multi-oscillatory and follow seasonal, ovulatory, and circadian cycles and are mediated through both the hypothalamic–pituitary–gonadal and hypothalamic–pituitary–adrenal axes, where communication is bidirectional [119–121]. Importantly, sex differences can contribute to variance of rhythms, for instance, in body temperature [122] and pubertal development [123], which should encourage inclusion of sex as a biological variable in circadian experiments.

Rhythms in the reproductive system can affect the susceptibility to pathogens that may impact fertility and reproductive health. Circadian disruption caused higher bacterial load, more pathology, and lower fertility rate in mice infected with *Chlamydia muridarum* [124]. Mice infected at the beginning of their rest phase shed more *Chlamydia* and were less fertile compared to infection at the beginning of the active phase [125]. Interestingly, different times of infection translated into differences in the vaginal, uterine, and ovary/oviduct microbiome, which were maintained even multiple weeks post-infection [126]. It is likely that these diurnal changes in the genital tract microbiome extend to infections of other pathogens and further research is warranted.

The circadian system can influence rhythms in the urinary tract, including bladder function [127] and kidney physiology. Renal plasma flow, glomerular filtration rate, and tubular reabsorption/secretion processes were shown to be rhythmic and peak during the active phase [128]. Despite limited research on the circadian regulation of pathogens infecting the urinary tract, findings suggest an impact on *Candida albicans* in the kidney [129]. Mice infected during the night exhibited reduced kidney colonization and weight loss, along with an increased survival rate compared to those infected during the day. This regulation was attributed to the neutrophil-intrinsic circadian clock and may extend to other kidney-infecting pathogens. *C*. *albicans* can infect various other organs, including the genitalia, which may also be subject to circadian regulation and warrants further investigation. This suggests that circadian immune processes may play a role in protecting against fungal infections, which is an intriguing and largely understudied topic for future research.

### The brain

Considering that the SCN in the hypothalamus serves as the central clock [130], it is intriguing to explore how pathogens influence the brain, even though there are constraints related to the blood–brain barrier.

Early studies show that herpes simplex virus 1 can invade the suprachiasmatic nuclei in mice with depleted CD4+ and CD8+ T cells, suggesting a potential mechanism to disrupt the circadian clock [131]. Additionally, during advanced stages of *T*. *brucei* infection, parasites and inflammatory cells can infiltrate the brain, which causes the characteristic symptoms of altered sleep and impairment of circadian-regulated processes [132–134]. Another pathogen crossing the blood–brain barrier is Zika virus, and viral replication is regulated by REV-ERBα/β through lipid signaling pathways [46]. However, these experiments were performed in a liver cell line, and the importance for brain-specific Zika virus infections remain to be elucidated. Encephalitis can be induced by vesicular stomatitis virus, and infection of mice at the beginning of the rest period caused higher mortality and increased inflammatory response compared to infection in the active cycle. This is likely mediated through a clock-regulated inflammatory response, as REV-ERBɑ inhibition at the start of the active phase abolished the phenotype [135].

It is worth considering the role of circadian rhythms on other neurotrophic pathogens, many of which can establish chronic infection and alter sleep rhythms, as demonstrated for *Toxoplasma gondii* [136]. Interestingly, crosstalk between the brain and daily changes in gut microbiota was shown to contribute to aging-associated neurodegenerative diseases [137]. However, how the microbiota–gut–brain axis and other pathogens interplay with brain circadian rhythms requires further investigation.

In summary, these examples of pathogen adjustments to the host niches underscore the significance of taking tissue-specific circadian control into account. It is likely that these extend even further to tissues that have not been studied in the circadian-pathogen context yet. For instance, it is interesting to speculate about the circadian rhythms of adipose tissue [138,139], which is a major reservoir of clock-regulated *Trypanosoma* parasites [91,140]. In addition to different environments within 1 host, it is fascinating how pathogens can adapt to various intermediate hosts and vectors [84,141]. Changes in cycling genes among different pathogen phases suggest the capability to adjust to diverse host settings, a characteristic feature of circadian clocks.

## 4. Therapeutic insights and future directions

There are several strategies for utilizing the insights on circadian pathogen regulation to enhance therapy [142–145]. Administering medication or vaccines at specific times can strategically target the pathogen when it is most vulnerable and optimize the immune response at the right time of day [115,146].

The discovery that many pathogens have their own internal clocks could pave the way for novel treatment strategies [20]. If the pathogen’s clock regulators are different from the host’s, targeting the pathogen’s clock could result in a “jet-lagged” microorganism, making it more vulnerable to elimination by host defense mechanisms. It could provide a targeted approach without collateral disruption of the host clock; however, the feasibility of selectively manipulating the pathogen’s clock remains unclear, and our understanding of pathogen-intrinsic clocks is still limited, hindering the design of such compounds.

Another strategy is to pharmacologically modulate the circadian system of the host, using compounds that target clock transcription factors. This has the limitation that these drugs may exert systemic effects on various biological rhythms and pathways, leading to off-target effects. Nonetheless, combining broad circadian modifiers with pathogen-specific drugs could offer a more targeted approach. A better understanding of organ-specific and host–niche interactions between circadian rhythms and pathogens will help to refine and characterize these strategies.

A topic that has received limited attention is the interplay between circadian rhythms and coinfections involving multiple pathogens. These infections frequently involve pathogens replicating in the same host niche due to a locally compromised immune defense, facilitating the infection by other pathogens. This is particularly significant as it can lead to more severe outcomes. Exploring approaches to selectively target circadian clocks within specific host niches could provide a novel and efficient way to target multiple pathogens. Although this concept is in its early stages, the comprehensive understanding of the interplay between pathogens and circadian clocks across various host environments can guide future studies. We hope that this review provides a holistic view on this intricate relationship and stimulates a new perspective on circadian pathogen research.
